# Molecular dissection of engraftment in a xenograft model of myelodysplastic syndromes

**DOI:** 10.18632/oncotarget.24538

**Published:** 2018-02-20

**Authors:** Mathieu Meunier, Charles Dussiau, Natacha Mauz, Anne Sophie Alary, Christine Lefebvre, Gautier Szymanski, Mylène Pezet, Françoise Blanquet, Olivier Kosmider, Sophie Park

**Affiliations:** ^1^ University Clinic of Hematology, CHU Grenoble Alpes, Grenoble, France; ^2^ TIMC-Therex, UMR 5525 CNRS, Grenoble Alpes University, Grenoble, France; ^3^ Hématologie Biologique, Hôpital Cochin, Assistance Publique-Hôpitaux De Paris, Paris, France; ^4^ Laboratory of Hematology, Onco-Genetic and Immunology, Biology and Pathology Institute, Grenoble, France; ^5^ Optical Microscopy and Flow cytometry Core, Institute for Advanced Biosciences, Inserm U 1209, CNRS UMR 5309, Université Grenoble Alpes, Grenoble, France; ^6^ Plate-Forme De Haute Technologie Animale, UMR5525, Grenoble Alpes University, Grenoble, France

**Keywords:** myelodysplastic syndrome, patient derived xenograft, bone marrow microenvironment, mesenchymal stromal cells, mutational hierarchy

## Abstract

Myelodysplastic syndromes (MDS) are oligoclonal disorders of the hematopoietic stem cells (HSC). Recurrent gene mutations are involved in the MDS physiopathology along with the medullar microenvironment. To better study the heterogeneity of MDS, it is necessary to create patient derived xenograft (PDX).

We have reproduced a PDX model by xenografting HSC (CD34^+^) and mesenchymal stromal cells (MSC) in NOD/SCID/IL2rγ^-/-^ mice with primary samples from one RAEB2, two RAEB1 and one RARS patients harboring karyotype abnormalities and gene mutations. The average human chimerisms ranged from 59.7% to 0.0175% for the 4 patients. Secondary grafts (G2) were only performed for mice derived from the RAEB2 patient and the average human chimerism was 53.33%. G1 mice 1 and 2, and their derived G2 mice showed less than 20% of medullar blasts whereas mouse 3 and the resulting G2 mice transformed to AML. Clonal architecture was dissected in the different hematopoietic progenitors (HP) harvested from G1 and G2 mice. By direct Sanger sequencing, we found the 4 initial mutations in each HP subpopulation and those mutations had the same variant allele frequency in the CD34^+^ CD38^-^ HSC from G1 and G2 mice by next generation sequencing (NGS). Targeted NGS analysis done in HSC of mouse 3 did not show any additional driver gene mutations explaining the transformation to AML.

To conclude, we have generated a PDX mouse model that perfectly reproduces the MDS founder clone which is stable over time, allowing us to consider this system as a powerful tool to test therapeutic approaches.

## INTRODUCTION

Myelodysplastic syndromes (MDS) are oligoclonal disorders of the hematopoietic stem cells (HSC) and are propagated by rare human MDS propagating cells [1, 2]. MDS comprise diverse phenotypes characterized by ineffective hematopoiesis leading to cytopenia, bone marrow dysplasia and transformation to acute myeloid leukemia in 30 to 40% of the cases [3].

Somatic mutations are common in MDS physiopathology and different functional groups are identified: splicing factors, epigenetic regulators, transcription factors and kinase signaling [4-7]. Moreover, the medullar microenvironment especially represented by the mesenchymal stromal cells (MSC) is also implicated in the development and propagation of dysplastic hematopoiesis [8-10]. Extensive data suggest that MDS-derived MSC are functionally altered and harbored epigenetic alterations [11]. To better study the heterogeneity of MDS and to develop new therapeutic drugs, it becomes necessary to create patient derived xenograft (PDX) mice models by engrafting CD34^+^ cells from MDS patients. Recent studies demonstrated that intramedullary co-transplantation method with HSC and MSC could improve engraftment on MDS in xenograft models [12]. Here, we describe an efficient PDX model by using primary CD34^+^ and autologous MSC from MDS patients. This model is stable over generations of mice and allows us to decipher the mutational hierarchy amongst different populations of early progenitors generated in mice by longitudinal next generation sequencing. We will use this tool for new therapeutic approaches.

## RESULTS

### Engraftment of patient-derived myelodysplastic syndrome in NSG mice

**Figure 1:**
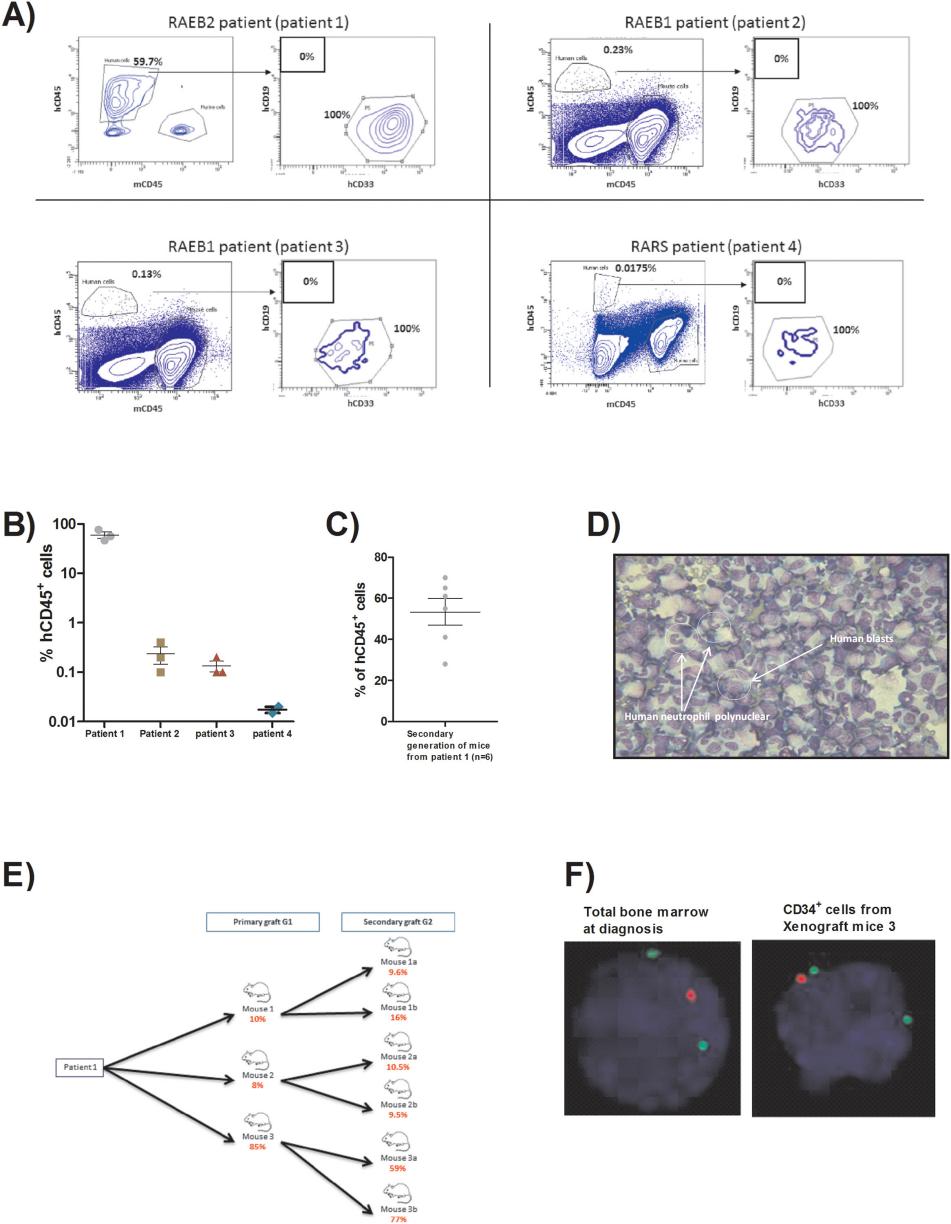
**(A)** FACS plots showing the percentage of human cells at sacrifice (6 months after transplant) for the four patients engrafted. **(B)** Scatter plot representing the percentage of human CD45^+^cells assessed by flow cytometry within the bone marrow retrieved from 1^st^ generation mice for each patient (6 months after transplant). **(C)** Scatter plot representing the percentage of human CD45^+^ cells assessed by flow cytometry within the bone marrow retrieved from 2^nd^ generation mice deriving from patient 1 (6 months after transplant). **(D)** Picture of bone marrow cytospin stained by MGG (x40) from 1^st^ generation mouse of patient 1 showing the human blast infiltration and low granularity of granulocytic lineage. **(E)** Flow chart representing the number of human blasts counted on bone marrow cytospin from each mouse of 1^st^ and 2^nd^ generations derived from patient 1 (6 months after transplant). **(F)** FISH detection of del 5q. Nucleus is stained in blue (DAPI), del5q probe in red and control probe in green (x63). Experiment done on total bone marrow from patient 1 at diagnosis and on CD34^+^ sorted cells from one mouse of 1^st^ generation (6 months after transplant).

We transplanted intramedullary CD34^+^ cells with autologous mesenchymal stromal cells from bone marrow obtained from patients with MDS in NOD/SCID/IL2rγ^−/−^ (NSG) mice with a ratio of 1/3 respectively for CD34^+^ and MSC [12]. Twenty-four hours prior to transplantation, mice received one busulfan injection intraperitoneally (25mg/kg). There were one refractory anemia with blast excess (RAEB) 2, two RAEB 1 and one refractory anemia with ring sideroblast (RARS) with karyotype abnormalities and recurrent gene mutations involved in MDS (characteristics are described in Table 1). Each patient sample gave rise to three mice. Six months after transplant, mice were euthanized as already described in Medyouf et al’s work [12]. No mice showed earlier signs of the disease like loss of weight or nose and eye bleeding. The bone marrow was investigated for human chimerism by flow cytometry based on human CD45, human CD33, human CD19 and murine CD45. The average human chimerisms (based on percentage of human CD45 positive cells) were respectively 59.7% (50.92 - 68.48); 0.23% (0.142 - 0.318); 0.13% (0.097 - 0.318) and 0.0175% (0.015 – 0.019) for the 4 patients (Figure 1A, 1B). As already described in previous studies, we have chosen a threshold of 0.01% of human cells to define a human engraftment in mice [13] and according to this threshold all the mice were engrafted. There was a clear myeloid bias in this human engraftment as we did not find any B positive cells.

**Table 1:** Table representing the principal biological characteristics of patients used for the xenograft model

	MDS	Percentage of medullary blasts at diagnostis	Karyotype	Molecular biology
**patient 1**	RAEB-2	14	46,XY,del(5)(q13q34)[11]/46,sl,add(18)(q21)[9]	TP53 (G245D), SF3B1(R625H), RUNX1 (R201Q) and KIT (D816V)
**patient 2**	RAEB-1	4	44-45,XY,add(3)(p12),-7,add(17)(p12),mar,min [2]/46,XY [18]	TP53 (G245D)
**patient 3**	RAEB-1	6	45,X,-Y,del(11)(q21q25)[1]/45,sl,ins(4;3)(q2?6;q28q24)[2]/90,idemx2 [1]/45,sdl1,dic(11;?)(q13;?)[12]/45,sl,t(6;12)(q25;q12),add(11)(q13)[4]/46,XY [2].ish ins(4;3)(EVI1+)	TET2 (R1216X)
**Patient 4**	RARS	1,5	46,XX [20]	SF3B1 (K666R)

RAEB: Refractory anemia with excess of blast; RARS: Refractory anemia with ring sideroblasts.

We also determined if the human mesenchymal stromal cells injected with CD34^+^ were still present at the end of the xenograft procedure. By flow cytometry and using a consensual panel of antibodies (CD90, CD73, CD105 and CD45) [14], we did not find any human MSC in mice bone marrow (data not shown) at 6 months of engraftment suggesting that MSC were essentially involved at the beginning of the engraftment process.

Secondary grafts were only performed for mice derived from patient 1 (RAEB2) and each mouse of the first generation gave rise to two mice for the second generation. These secondary grafts were performed by intra bone injection of 1x10^5^ CD34^+^ cells without autologous MSC to assess the propagating capacity of the cells obtained after the first PDX. For the secondary graft, we hypothesized that the engraftment would be faster so we performed a tibia bone marrow aspiration to monitor the engraftment 3 months after injection and as human engraftment was already evidenced by this intermediate point, the mice were sacrificed at 3 months. The average human chimerism for the six mice of the second generation was 53.33% (36.61 - 70.05), not different from the chimerism obtained at the first engraftment (Figure 1C).

### Molecular dissection of the bone marrow compartment of mice engrafted with myelodysplastic syndrome-originated cells

Then, we focused on biological materials from patient 1 graft for all further experiments. First, morphological observations of bone marrow cytospins stained by May-Grünwald Giemsa (MGG) coloration of engrafted mice from first and second generations showed presence of human blasts and human neutrophils and characteristics features of dysplasia already described in the original patient sample (Figure 1D). Moreover, we have realized human blasts count on those cytospins. Interestingly, mice 1 and 2 showed less than 20% of medullar blasts and also for their derived 2^nd^ generation mice (10.6% (9.47 – 11.73)) whereas mouse 3 and the resulting secondary graft mice harbored more than 20% of medullary blasts (73.67% (65.98 – 81.35)) suggesting a more aggressive disease (Figure 1E).

We performed FISH analyses of CD34^+^ cells from the first generation of mice and found the same cytogenetic abnormality (del5q) in 87.5% of the CD34^+^ cells harvested from the mice *versus* 79% in total bone marrow cells at diagnosis of the patient 1 (Figure 1F).

**Figure 2:**
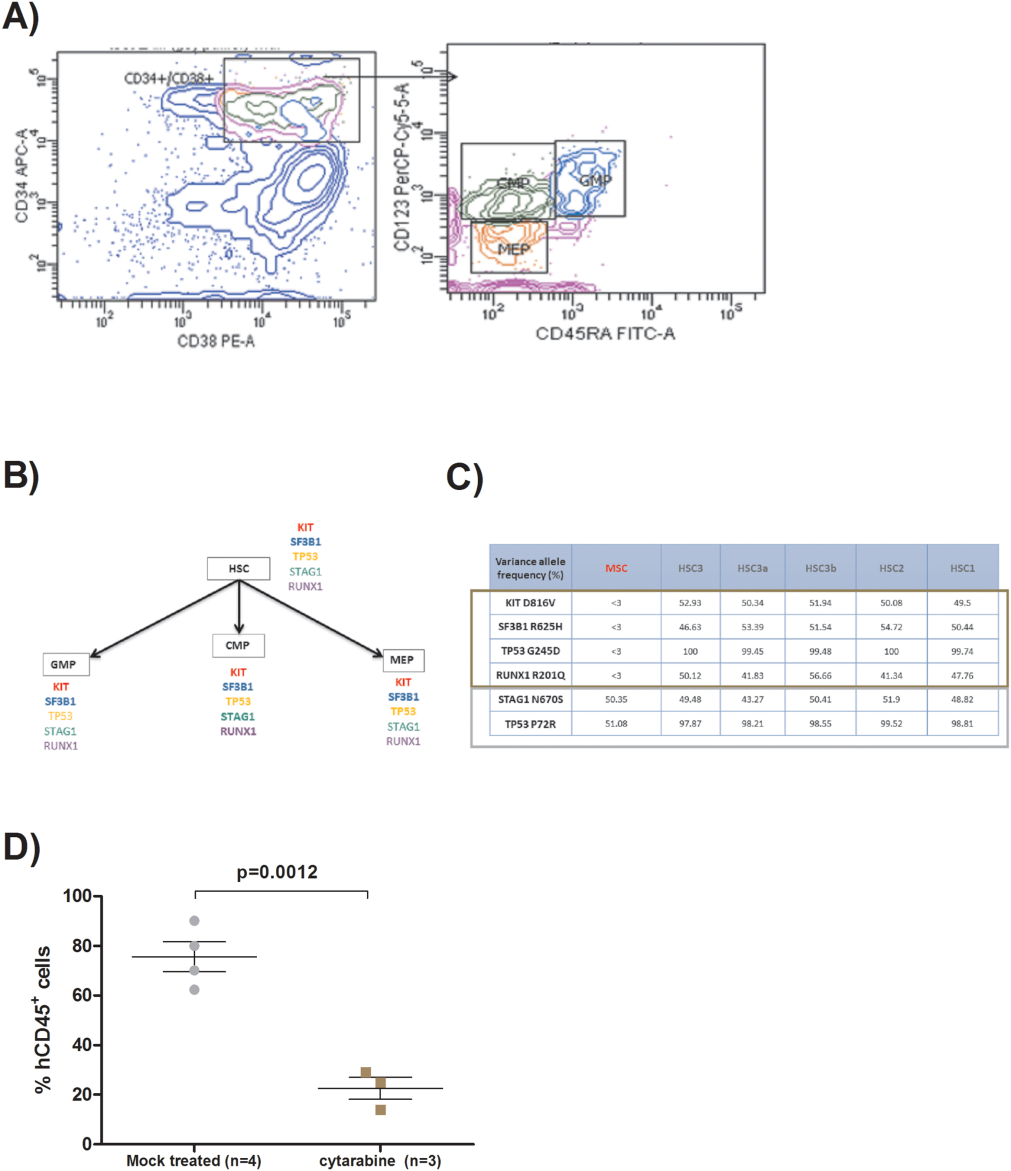
**(A)** FACS plots showing the method used for hematopoietic progenitors sorting from xenografted bone marrow. Hematopoietic stem cells (HSC) are CD34^+^/CD38^-^, common myeloid progenitors (CMP) are CD34^+^/CD38^+^/CD123^+^/CD45RA^-^, granulocyte macrophage progenitors (GMP) are CD34^+^/CD38^+^/CD123^+^/CD45RA+, and megakaryocyte–erythroid progenitors (MEP) are CD34^+^/CD38^+^/CD123^-^/CD45RA^-^. Human cells were previously sorted with human CD34^+^ magnetic beads. **(B)** Flow chart describing the presence of the different gene mutations in each kind of human progenitors sorted from the bone marrow of the different generations of mice derived from patient 1. **(C)** Table representing the different variant allele frequency obtained by next generation sequencing on hematopoietic stem cells (HSC) and mesenchymal stromal cells (MSC) from patient 1 xenografted mice. The brown rectangle represents the somatic mutations and the grey one polymorphisms. **(D)** Scatter plot representing the effect of cytarabine on the percentage of human CD45^+^cells assessed by flow cytometry within the bone marrow retrieved from xenografted mice deriving from patient 1. Two groups, one with mice treated with cytarabine intraperitoneally (10mg/kg) 5 days/7 during two weeks and another group with mock treated mice.

We then isolated hematopoietic stem cells (HSCs), common myeloid progenitors (CMPs), granulocyte macrophage progenitors (GMPs) and megakaryocyte–erythroid progenitor (MEPs) by FACS cells sorting based on specific antigens (CD34, CD38, CD45RA and CD123) as already described [15] (Figure 2A) from xenografted bone marrow from first and second generations of mice. We performed targeted sequencing for genes with initial mutations found in the patient 1 sample (KIT, SF3B1, TP53 and RUNX1) in all the progenitors by Sanger technique with specific primers. We found these initial mutations in all the different progenitors in 1^st^ and 2^nd^ generation of mice (Figure 2B). We performed also next generation sequencing (NGS) on a panel of 39 genes previously described and involved in MDS physiopathogenesis [16] in the HSC from the first-generation mice to check if the xenograft procedure could induce other mutations than specified in the initial sample of the patient 1, and in HSC derived from mouse 3 to analyze if the excess of blasts number, reflecting probably a more aggressive disease, was associated with new mutations. Concerning HSC, the variant allele frequencies (VAF) of initial mutations were similar between all the mice from the first generation and mice derived from mice 3 (45-50%) (Figure 2C). No new recurrent mutations were found to explain the higher blasts level of mice 3 and its derived mice. NGS was also performed on the initial mesenchymal stromal cells used during the xenograft procedure. Neither the four initial mutations nor other known recurrent mutation in MDS were found in these MSC. We found two polymorphisms, one well-known on *TP53* gene and another one on *STAG1* gene in both HSC and MSC, strongly suggesting their germinal origin.

### Chemotherapy validation of the model

Furthermore, we validated our model by using a therapeutic approach with chemotherapy. Indeed, we have treated engrafted mice with intraperitoneally injection of cytarabine (10mg/kg) five days per week during two weeks at the third month post-transplant. The mice were killed one week after the end of cytarabine course. Bone marrow chimerism was analyzed as previously described. Mock treated mice show an average 75.65% (69.4 – 81.66) of human CD45^+^ cells *versus* 22.67% (18.69 – 27.15) (p=0.0012) for mice treated with cytarabine (Figure 2D). We have also done cell sorting for the HSCs, CMPs, GMPs and MEPs in order to perform targeted sequencing for initial gene mutations. These mutations were found in all the progenitors, reflecting the fact that although cytarabine reduces human chimerism, all the mutated progenitors remain and will probably be the stem of future relapses.

## DISCUSSION

We demonstrate here the possibility to make a PDX mouse model that perfectly reproduces the MDS founder clone. We chose to add MSC to CD34^+^ at the first generation of mice as we believe as Medyouf et al [12], that a humanized microenvironment could improve engraftment of MDS CD34^+^ cells. This method allowed us to get an engraftment success of 100% of our patients although the series is small. There is controversy about the beneficial effect on engraftment of adding mesenchymal stromal cells to CD34^+^ cells [13]. Muguruma et al have [17] achieved a successful engraftment of myelodysplastic syndrome by adding MSC to CD34^+^ cells but the efficiency of the engraftment was related to the presence of an abnormality in chromosome 7. Li et al [18] obtained better chimerism by engrafting CD34^+^ cell with a human marrow stroma cell line (HS27a). Those results were confirmed by Medyouf et al [12] with direct intrabone injection of CD34^+^ cells and MSC. Like Medyouf et al, we have also chosen some higher risk MDS patients with RAEB1 and RCMD with complex karyotype. Nevertheless patient 1 had a more aggressive disease.

Recently, Rouault-Pierre et al [13] showed that co-injection of CD34^+^ cells with MSC did not improve the level of engraftment in a cohort of 10 MDS samples. These data suggest that engraftment potential of the MDS CD34^+^ did not depend on the presence of human MSC. Interestingly, in Medyouf’s study, the secondary grafts were performed without adding MSC and cells successfully engrafted as we have found. We can hypothesize that MSC cells could improve the level of engraftment for primary graft, but for secondary graft, the propagation capacities of the sole cells are sufficient to engraft.

In one patient, all the mutations were present very early in the course of the disease in the HSC, CMP, MEP and GMP. This clone is very stable over time as it does not disappear in the second generation of mice and no new mutations were found neither. Recently, Belderbos et al [19] demonstrated in a xenograft model of lymphoblastic acute leukemia that serial transplantation is leading to a clonal selection with a deterministic pattern. In our model, it seems that serial transplantation increases the potential of reconstitution of the LSC, as we transplanted a lower number of CD34^+^ in the 2^nd^ generation of mice, but obtained an increased number of blasts in mouse 3, but this was not reflected by a clonal skewing or expansion in the sequential HSC sequencing in our MDS model.

The mutations were still present in the minimal residual disease after cytarabine too, confirming that traditional chemotherapy does not reach the leukemic stem cells and just reduces the bulk blasts. Therefore, we generated a PDX model of MDS where the clonal architecture was not altered. This *in vivo* model represents an interesting tool to define therapeutic approaches in high-risk MDS models.

## MATERIALS AND METHODS

### CD34^+^ and mesenchymal stromal cells (MSC) selection from primary samples

Bone marrows from myelodysplastic patients were collected by bone marrow aspiration after signed consent and approval by our local ethical committee. By density gradient using Ficoll (Eurobio), the mononuclear cells were isolated from total bone marrow. HSPCs (hematopoietic stem progenitor cells) were positively selected by paramagnetic iron-dextran particles directly conjugated to anti-CD34 monoclonal antibodies (CD34 MicroBead Kit, human, Miltenyi Biotec) by using an automatized system (MACS pro, Miltenyi Biotec). Their viability was assessed by trypan blue exclusion (Sigma Aldrich). The purity of all samples was checked by flow cytometry (BD FACS Canto II, BD Biosciences) with a specific CD34 fluorochrome-conjugated antibody (CD34-APC, Miltenyi Biotec) and was superior to 90% for all samples used. The same procedure was used for CD34^+^ cells sorting from xenografted mice bone marrow.

The CD34 negative fractions or total bone marrows from MDS patients were incubated in culture flask of 75cm^2^ (BD Falcon™) with DMEM (Dulbecco’s modified eagle medium, Life technologies, Paisley, UK) plus Fetal serum bovine (ThermoFisher Scientific) at 10%, antibiotics (penicillin and streptomycin, Life technologies™) and L.glutamine at 1% (Glutamax, ThermoFisher Scientific) at 37°C. Mesenchymal stromal cells were isolated by adherence to plastic after multiple washes by phosphate buffering saline, and their phenotype was confirmed by flow cytometry (BD FACS Quanto II, BD Biosciences) using CD73 (CD73APC, Miltenyi Biotec), CD90 (CD90PE-CY™ 7, BD Biosciences), CD105 (CD105PE, Miltenyi Biotec) and CD45 (CD45V500, BD horizon™, BD Biosciences). All MSC were positive > 95% for CD73, CD90 and C105 and negative for CD45. Moreover, we have checked their ability for trilineage adipogenic, osteogenic and chondrogenic differentiation, meeting the conventional criteria for MSC [20]. Osteoblastic, adipogenic and chondrogenic differentiation medium used were commercial solutions (StemMACS™ OsteoDiff Media, StemMACS™ AdipoDiff Media, StemMACS™ ChondroDiff Media, MACS, Miltenyi Biotec). Culture medium were replaced every 4 days for 21 days. Then osteogenic differentiation was evaluated by alkaline phosphatase staining (SIGMA FAST™ 5-Bromo-4-chloro-3-Indonyl phosphate, Sigma-Aldrich), adipogenic differentiation by staining with Oil Red O (Abcam®) and chondrogenic differentiation by sulfated proteoglycans staining with alcian blue (Leica Biosystems) (Supplementary Data, Figure 1). All the MSC used for the xenograft model were inferior to passage 5.

### Xenotransplant assays

NOD/SCID/IL2rγ^−/−^ (NSG) mice were maintained in sterile isolator cages in the animal facility of the Grenoble Alpes University. NSG mice used in this study were 8 to 12 weeks old at the beginning of experiments. Twenty-four hours prior to transplantation, mice received one busulfan injection intraperitoneally (25mg/kg). Direct intra bone marrow injection was performed in the tibia with 5x10^5^ CD34^+^ cells and 1.5x10^6^ MSC from the same patient. Prior to injection, CD34^+^ cells were thawed. Secondary grafts were performed by intra bone injection of 1x10^5^ CD34^+^ cells. The mice were monitored everyday according to a specific wellness scoring. The mice were weighted every week and in case of bleeding or asthenia, a retro-orbital blood analysis was done. For the secondary graft, we hypothesized that the engraftment would be faster so we performed a tibia bone marrow aspiration to monitor the engraftment 3 months after injection. All procedures were approved by the Animal care committee of Grenoble Alpes University (agreement number: B385161006).

### Human chimerism and morphological analyses

The mice were killed at the end of the xenograft procedure (6 months). Bone marrow contents from tibiae, femora and humeri were collected. The human chimerism was determinated using FACS flow (quanto II, BD) with the following antibodies human CD45 (CD45-Viogreen™, Miltenyi Biotec), murine CD45 (CD45 PeRCP-Vio700, Miltenyi Biotec), human CD33 (CD33-PE, Miltenyi Biotec) and human CD19 (CD19-APC, Miltenyi Biotec). As already described in previous studies, we have chosen a threshold of 0.01% of human cells to define a human engraftment in mice [13]. Aliquot of each bone marrow were stained with May-Grunwald-Giemsa (MGG) by an automatized way (Aerospray^®^Pro, Elitech). Then, the % of human’s blasts were counted and the morphology of the cells was described by a trained cytologist.

### Fluorescence *in situ* hybridization

For detection of the 5q deletion, FISH targeting the EGR1 gene was performed on CD34^+^ sorted cells and total bone marrow using a dual-color probe (Del(5q) deletion Cytocell® : EGR1-Tx-Red/control probe 5p15-FITC). Briefly, slides were fixed in methanol/acetic acid 3:1 for 30 minutes, air-dried, and dehydrated through graded alcohols. Both FISH probes and target DNA were denatured simultaneously for 3 minutes at 75°C and incubated for 20 hours at 37°C. The post hybridization washes were performed according to the manufacturer’s instructions (Cytocell®). Two hundred nuclei were scored.

### Isolation of MPPs, MLPs, CMPs, GMPs and MEPs

CD34^+^ human cells obtained at the end of the xenograft procedure were stained with the following antibodies: CD34 (CD34-APC, Miltenyi Biotec), CD123 (CD123-PerCP-CY5.5, BD Bioscineces), CD45RA (CD45RA-FITC, IOTest^®^) and CD38 (CD38-PE, Miltenyi Biotec). Hematopoietic stem cells (HSCs), common myeloid progenitors (CMPs), granulocyte macrophage progenitors (GMPs) and megakaryocyte–erythroid progenitor (MEPs) were isolated as already described [15]. DAPI (Thermo Scientific) was added to the cell suspension to exclude dead cells. Cells were sorted on a BD FACS Aria (San Jose, CA) operating in 4 way purity sort mode, and collected into 5 ml polypropylene tubes. Then the cells were washed in phosphate-buffered saline solution.

### DNA isolation

The method used for DNA isolation differed with the number of cells. For more than 100 000 cells, and between 100 000 and 10 000 cells, the QIAmp DNA Mini Kit (QIAGEN) and the FlexiGene DNA Kit (QIAGEN) were used respectively following the manufacturer’s instruction. For less than 10 000 cells, cells were lysate with tween 20 and proteinase K.

### Sanger and next generation sequencing

A selected panel of 39 genes recurrently mutated in MDS was designed and mutations were identified by next-generation sequencing (NGS) in BM mononuclear cells (MNCs) using a PGM system (ThermoFisher Scientific, Waltham, MA) and the 384-reaction Ion AmpliSeq library kit 2.0 (Life Technologies, Chicago, IL) Base calls were generated by the Torrent Browser software (Life Technologies). The annotated.vcf files were also analyzed using NextGENe software (Softgenetics, State College, PA, USA). Abnormalities identified by both analyses were considered if referenced in UCSC and absent in 1000 genomes databases. For a given mutation, the proportion of mutated cells was twice the VAF of heterozygous mutations, or one-fold the VAF for homozygous mutations or hemizygous mutations of X chromosome genes in males) [21].

### Statistics

The significance of the experimental results was determined with an unpaired Student test. p≤0.05 was considered statistically significant. All statistical analyses were done with GraphPad Prism 5 software (http://www.graphpad.com/scientific-software/prism/). Data are mean with SEM.

## SUPPLEMENTARY MATERIALS FIGURE


