# Supplementary material for: Molecular dissection of engraftment in a xenograft model of myelodysplastic syndromes
Source: Oncotarget. 2018 Feb 20;9(19):14993–5000. doi: 10.18632/oncotarget.24538 (PMC5871091; doi:10.18632/oncotarget.24538)
Supplement: Supplementary file 1 [file oncotarget-09-14993-s001.pdf]

Molecular dissection of engraftment in a xenograft model of myelodysplastic syndromes

SUPPLEMENTARY MATERIALS

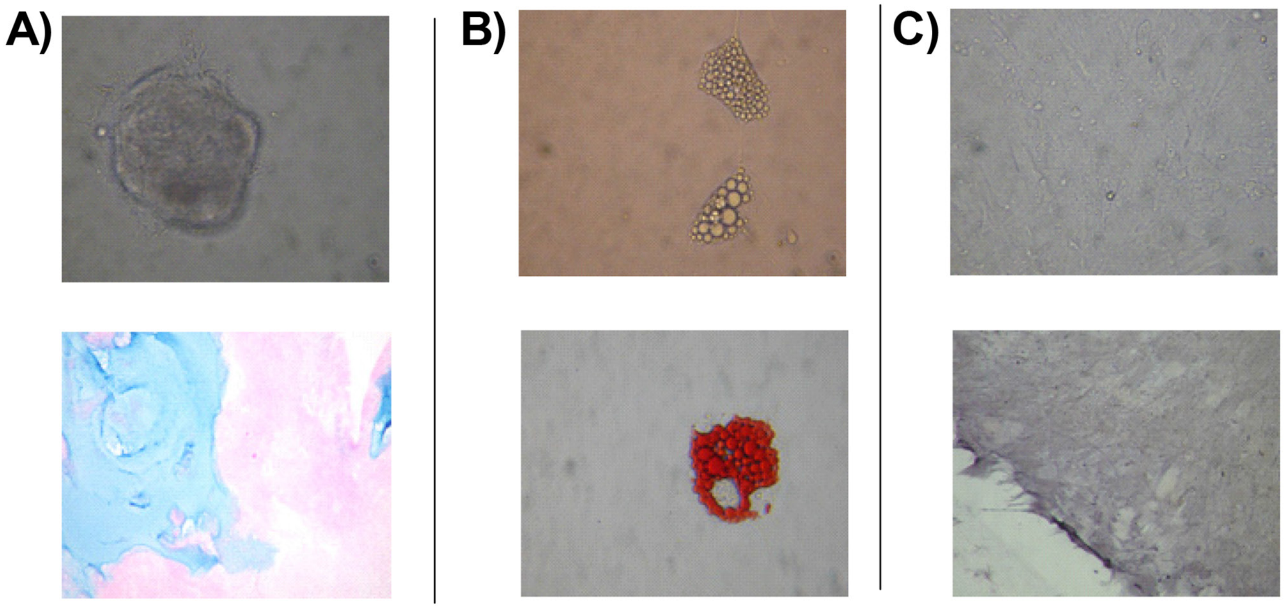

Supplementary Figure 1: Results confirming the ability for trilineage differentiation of mesenchymal stromal cells (adipogenic, osteogenic and chondrogenic). **(A)** Chondrocyte before and after alcian blue staining, X400. **(B)** Adipocyte before and after red oil staining, X400. **(C)** Osteocyte before and after alkaline phosphatase staining, X40. All the methods are described in the manuscript.
